# Cloud-Based Monitoring of Thermal Anomalies in Industrial Environments Using AI and the Internet of Robotic Things

**DOI:** 10.3390/s20216348

**Published:** 2020-11-07

**Authors:** Mohammed Ghazal, Tasnim Basmaji, Maha Yaghi, Mohammad Alkhedher, Mohamed Mahmoud, Ayman S. El-Baz

**Affiliations:** 1 Electrical and Computer Engineering Department, College of Engineering, Abu Dhabi University, Abu Dhabi 59911, UAE; tasnim.basmaji@adu.ac.ae (T.B.); maha.yaghi@adu.ac.ae (M.Y.); 2 Mechanical Engineering Department, College of Engineering, Abu Dhabi University, Abu Dhabi 59911, UAE; mohammad.alkhedher@adu.ac.ae; 3 Emirates Global Aluminium, Technology Development and Transfer Midstream, Abu Dhabi 109111, UAE; mmahmoud@ega.ae; 4 Bioengineering Department, University of Louisville, Louisville, KY 40292, USA; ayman.elbaz@louisville.edu

**Keywords:** edge-fog-cloud computing, Internet of Things, robotics, artificial intelligence, autonomous driving, image registration

## Abstract

Recent advancements in cloud computing, artificial intelligence, and the internet of things (IoT) create new opportunities for autonomous industrial environments monitoring. Nevertheless, detecting anomalies in harsh industrial settings remains challenging. This paper proposes an edge-fog-cloud architecture with mobile IoT edge nodes carried on autonomous robots for thermal anomalies detection in aluminum factories. We use companion drones as fog nodes to deliver first response services and a cloud back-end for thermal anomalies analysis. We also propose a self-driving deep learning architecture and a thermal anomalies detection and visualization algorithm. Our results show our robot surveyors are low-cost, deliver reduced response time, and more accurately detect anomalies compared to human surveyors or fixed IoT nodes monitoring the same industrial area. Our self-driving architecture has a root mean square error of 0.19 comparable to VGG-19 with a significantly reduced complexity and three times the frame rate at 60 frames per second. Our thermal to visual registration algorithm maximizes mutual information in the image-gradient domain while adapting to different resolutions and camera frame rates.

## 1. Introduction

Autonomous robots are smart machines capable of performing complex and repetitive tasks with a high level of independence from human intervention [1]. According to the International Federation of Robotics, industrial robots represent the largest percentage of all robots used and are expected to continue to be the leading robotic category until 2025 [2]. Recent advancements in AI and computer vision paved the way for robots to navigate challenging industrial environments for condition monitoring and assessment. They require less control and observation and can work efficiently and accurately [3]. Some of the reasons behind the challenging nature of industrial environments include the size of the monitored area, sensors cost, process effect on electronics and communications networks, obstacles, accessibility of monitored areas, high temperature, and low-lighting conditions. An example of challenging environments is industrial aluminum factories. In these factories, steel pot shell surfaces’ temperature needs to be regularly measured to analyze sick pots, amperage increase, and design changes in pot lining. The pots’ conditions must be continuously monitored and assessed, typically done manually by humans despite being unsafe and carrying a risk of exposure to harm. An alternative approach is to use the Internet of Things (IoT) architectures and install sensors-equipped nodes to cover the monitored area. Using IoT nodes provides an early response to anomalies and reduces the risk of injury. There are two main disadvantages to this approach when applied to some industrial settings including: (1) industrial processes may produce conditions which affect the electronics and communication capabilities of the IoT nodes (e.g., high temperatures or magnetic fields); (2) the cost of covering a large industrial area reduces deployment, especially as the cost of the sensors onboard increase.

To reduce or eliminate the need for human involvement in monitoring challenging industrial environments and reduce the cost of large-scale IoT nodes deployment, we propose mobile IoT nodes carried onboard autonomous robots. These robots must be designed to simultaneously address the application’s requirements and the challenges posed by the environment. In our example case of aluminum factories, the application requirements are to thermally monitor a sizeable industrial area without a large scale deployment of thermal-camera-equipped nodes. Instead, we are to use low-cost AI-powered robots to navigate and monitor the environment. The robot detects, localizes, and visualizes thermal anomalies within a short response time. The environment’s challenges include a strong magnetic field interfering with electronic circuits and virtually non-existent wired or wireless communication channels. The large scale installation of custom-designed communication solutions is also not feasible. The temperature and humidity are high, and the surface on which the robot will move is rough, with many obstacles to avoid. The system is to report the visual and thermal videos to a centralized repository for archiving and further short- and long-term automated analyses. Reports are to be available on mobile devices to facilitate access to them on-site. While we have used the aluminum pot-lines as a basis for our design and validation, our proposed solution can be used in other automated condition monitoring in challenging industrial environments after an initial setup stage which includes: (1) additional training for the self-driving sub-system; (2) thermal cameras and detectors re-calibration; and (3) placement of localization QR codes.

To address the requirements and constraints, we propose an edge-fog-cloud architecture for a low response time, larger covered area, and overall reduced deployment costs. The mobile IoT nodes at the edge layer comprise of multiple anomalies detecting autonomous robots. The robots initially use real-time global thresholding of thermal images for anomalies detection but cannot communicate the alarm to maintenance staff due to the lack of communication channels. The robot dispatches its drone with the thermal and visual images and coarse location data to leave the dead communication zone and deliver the alarm information as soon as it regains connectivity, thus maintaining a fast response time. We place the drones in the fog layer of our architecture, which, along with the robots, form an Internet of Robotic Things. The drones can also be used to initiate automated early response actions. When a robot run (i.e., a full area scan) is complete, their ability to communicate is restored by leaving the dead communication zone. The thermal and visual videos and location information are uploaded to a cloud server for registration, fine localization, and further analysis of the anomalies (e.g., anomaly classification or thermal distribution analysis).

The robot integrates high- and low-level sub-systems to perform its function, including obstacle avoidance, self-driving, localization, remote control, and anomaly detection and reporting. We propose a three-layered architecture for these sub-systems to govern their communication and coordination. In addition to the above contributions, we also propose a deep learning convolutional neural network for regressing the steering angle of differential robots that can deliver a root mean square error comparable to well-known architecture but with a faster frame rate and less computational complexity. Moreover, since the frame rates and image resolutions of the thermal and visual imagers are different, our cloud server needs to align and overlay the thermal to the visual video to help the end-users localize and analyze the detected thermal anomalies. To this end, we also propose a thermal to visual frame-rate and resolution adaptive multi-modal image registration technique based on maximizing mutual information in the image gradient domain.

The main contribution of our work is an end to end system for the autonomous monitoring of thermal anomalies in industrial settings. To achieve this goal, we also propose: (1) an autonomous robot surveyor operating system architecture for mobile IoT nodes; (2) an edge-fog-cloud communication architecture that reduces monitoring cost and anomalies detection time; (3) a real-time and accurate self-driving deep learning network; (4) a localization system based deep object detection of visual beacons; (5) a frame-rate and resolution adaptive thermal to visual registration algorithm based on maximizing mutual derivative information.

## 2. Literature Review

Several approaches in the literature propose autonomous robots for industrial applications. According to the authors in ref. [4], the modern industry is devoting efforts to the construction of unmanned factories, autonomous industrial robots, and the development of the digital twin techniques to eliminate potential human mistakes. The authors presented an industrial cyber-physical system monitoring and a control framework for large-scale hierarchical systems. The main purpose is to define the tasks to be performed at each stage of operation in modern large-scale industrial systems and the techniques that need to be applied to carry out these tasks. An autonomous vehicle used in smart factories was presented in ref. [5]. The system is based on HD mapping technology. The methodology involves real-time object detection and localization. Recognized objects are then compared with stored ones in the database to check their validity in the HD map and update them accordingly. The ATV can then use the updated map. Another use of such robots is for gas leakage detection, as proposed in ref. [6]. The robot introduced has two modes of operation: line following and obstacle avoidance. The robot is equipped with ultrasonic sensors for obstacle avoidance and an infrared sensor for leakage detection. Real-time leakage detection is based on the navigation through a dangerous environment and transmission of the intensity through a wireless signal to the receiving module for gas leakage monitoring. In ref. [7], the authors proposed the ARCO robot, which aims to work with laborers to collect material, facilitate transportation, and manage schedules in a factory. Similar to some of the previously discussed systems, the ARCO robot needs a map for localization purposes. However, the ARCO robot’s localization uses a previously generated map and computes a fixed map during operation. The robot has two modes of operation: straight-line and omnidirectional and includes an obstacle avoidance system for safety purposes.

A recent study in ref. [8] provides a comprehensive review of the main challenges proposed in the literature for autonomous robots in various environments. The challenges mainly include navigation and localization. Nowadays, some navigation systems involve lidar-based techniques for localization and mapping; however, such a technique does not perform well in smooth environments due to the lack of details and irregularities. On the other hand, Visual SLAM techniques are also used for localization in some systems. These techniques depend on features that describe the environment in terms of color and, therefore, need proper illumination, which becomes a problem in some environments like tunnels. To ensure better performance, multiple other systems rely on QR codes for navigation. The work in ref. [9] is a warehouse management robot that aims to automate tasks in an unknown environment. The robot is equipped with a robotic arm for handling items and a QR scanner for navigation and localization. It also involves a route development technique based on QR codes’ placement in strategic locations around the environment. The robot can extract location information for less computation and navigate to the required location from these codes. Recent systems developed in ref. [10,11] also use a similar indoor navigation system based on QR codes. A system developed in ref. [12] for assisting the elderly uses the Unscented Kalman Filter and QR codes for localization. The system provides the user with the ability to choose the desired location on the map and navigates autonomously to the chosen location. 

Several other papers focused on autonomous robots in industrial environments. Authors in ref. [13] developed an autonomous robot for mapping unfamiliar environments and identifying target objects. The methodology involves extracting and collecting a set of features during the setup stage. Accordingly, the robot will search for the features that match the stored features after developing a map using ultrasonic sensors and an RGB stream. In ref. [14], the authors proposed an autonomous robot used to monitor oil and gas locations. The robot operates in 4 modes: autonomous, manual, rail, and unsafe modes depending on the task. The rail mode enables the user to manually control the forward speed for manual inspections, where complicated operations are performed automatically. In comparison, the unsafe manual mode disables all safety features to operate in an unknown environment. The system also includes obstacle detection and lidar-based localization algorithms to drive the robot autonomously. Various approaches were proposed in the literature to solve the autonomous robot trajectory. The work in ref. [15] is an autonomous surveillance robot used to detect anomalies, including temperature instabilities and unauthorized personnel. The robot is equipped with multiple sensors, a near-infrared camera, and a Wi-Fi adapter. It navigates autonomously by collecting Lidar data and transmitting them to a central server for processing and map generation. The robot has an obstacle avoidance system to avoid any collisions with obstacles. The user needs to drive the robot manually in the environment to complete the mapping process in a semi-autonomous mode. Authors in ref. [16,17] used neural networks and artificial neural networks mainly for self-driving purposes. Whereas Deep and Convolutional Neural Networks were explicitly used in ref. [18]. 

Anomalies detection and recognition is typically one of the challenging requirements in industrial systems. An activity recognition system is proposed in ref. [19] designed for industrial surveillance systems. The framework proposed starts by using CNN-based human saliency features to select shots captured from surveillance videos. Temporal features are then extracted from the shots using the layers of the FlowNet2 CNN model to represent an activity. Accordingly, activities are recognized using a multi-layer long short-term memory. The proposed method achieved the highest detection accuracy results compared to previous methods for the Youtube activities dataset in terms of accuracy. The time complexity of the system is almost real-time making it suitable for industrial surveillance environments. Another framework is proposed in ref. [20] for similar environments. The framework aims to detect real-world anomalies in surveillance environments. The pipeline starts with extracting CNN features from frames and then inputting them to a multi-layer BD-LSTM for class detection. Compared to other anomaly detection methods, the proposed framework achieved higher accuracy and a low false alarm rate. Authors in ref. [21] presented a data-driven fault detection approach based on the integration of the modified principal component analysis (MPCA) into a locally weighted projection regression framework and compared it to several other approaches. The approach aims to monitor processes based on sensing measurements in complex nonlinear systems using the normalized weighted mean of test statistics. Depending on the modeling phase, different models are used to approximate the nonlinear systems. Then, the fault detection approach is applied for fault diagnosis. Compared to other approaches, the MPCA approach is less complicated, has lower computational complexity, a fast learning capacity, enhanced robustness, and a high fault detection rate. 

The remainder of the paper is organized as follows: Section 3 introduces the materials and methods. Section 4 discusses the testing and validation results of our proposed system and sub-systems. Finally, conclusions are drawn in Section 5.

## 3. Materials and Methods

### 3.1. Environment and Requirements

Automatic and on-time detection of machine anomalies in harsh industrial environments is essential for establishing reliable preventive or predictive maintenance schemes. The current manual diagnosis methods are limited to operator training capabilities, machine accessibility, and worker ability to endure the harsh industrial conditions [22].

Industrial environmental conditions are very harsh; this has restrained the use of electronics and communication systems in anomalies detection due to high temperatures, high humidity, dust, and magnetic fields. To improve normal practices in monitoring operating conditions of equipment and machines and overcome the harsh operating conditions, digitization of inspection processes is ramping up due to the availability of several sensing devices, data processing and storage capabilities using cloud computing, and affordable machine learning technologies. This has allowed maintenance protocols to utilize real-time sensing data to forecast possible anomalies and failures in machines due to several operating conditions and causes. Big data analysis is needed to study failure history and system performance that could require performing predictive maintenance. This analysis is powered by machine learning techniques to optimize available solutions.

Researchers have proposed using sensor data anomalies with statistical machine learning methods to predict machines’ unfavorable temperature in industrial manufacturing environments [23,24]. To improve the performance of anomaly detection systems in industrial facilities, machine learning has been implemented in predictive maintenance of industrial plants. These models are used to define the condition of the machines [25,26].

In aluminum production’s industrial environment, regular thermal measurements of the electrolytes take place in the basement of the potrooms to maintain heat balance and prolong the pot shell life cycle. The concept of the heat balance in the aluminum reduction pots is quite simple; almost half of the fed energy is actually used to produce aluminum. The remaining part must be dissipated as heat losses by the cell for it to maintain its thermal equilibrium. To maintain consistent pot performance, the thermal balance is regularly assessed during the whole pot life. These measurements are performed manually using thermocouples and thermometers at an ambient temperature that could reach 60 °C and humidity of 100% [27]. Moreover, sending human operators poses health and safety risks due to aluminum tap out and exposure to a high magnetic field of 300–500 Gauss.

Based on potline operation procedures, if the surface temperature of the potshell exceeds approximately 500 °C, the system operators consider that hotspot as an abnormal measurement. This detected thermal anomaly has to be monitored for any future excessive temperatures which could be caused by faults associated with the aluminum production process [28]. Regular potshell surface temperature measurements are performed at different locations to help in studying and analyzing potshell thermal balance. Detecting thermal anomalies will help operators to activate a cooling system immediately to maintain the current efficiency of the produced aluminum, identify damaged pots, and improve potline design [27].

**Figure 1:**
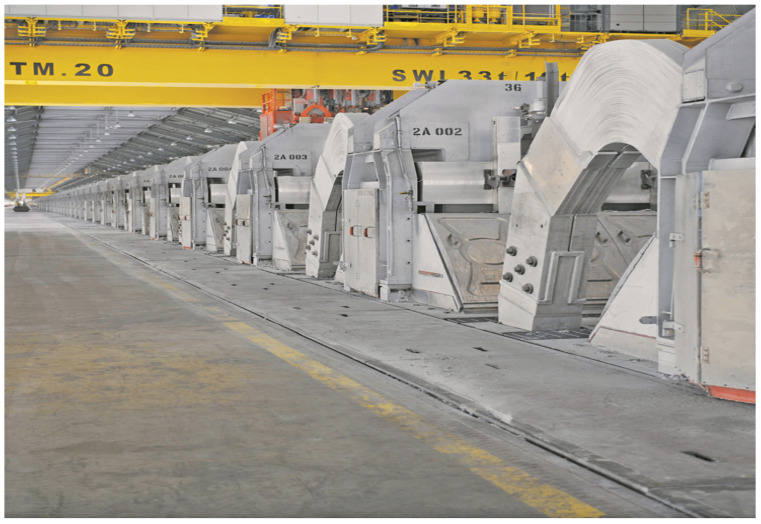
System’s working environment characterised by a high magnetic field up to 300–500 Gauss, ambient temperature up to 60 °C, high pot shell temperature varying between 120 °C and 500 °C, high relative humidity up to 100%, and concrete debris on the floor.

Pot shell temperature varies between 120 °C to 500 °C at standards conditions. The measurements can vary based on location and process parameters. In this work, the robot surveyor should monitor any excessive change above 500 °C. A regular inspection of potshell surface measurement is carried out every two-three weeks. Rapid detection of thermal anomalies can help operators and process engineers to timely respond to problems that occur in the process and avoid damage in the potshell. Figure 1 shows a depiction of the operating environment. Due to the characteristics of the working environment, the system is inaccessible to readers.

### 3.2. Proposed Robot Surveyor Operating System Architecture

**Figure 2:**
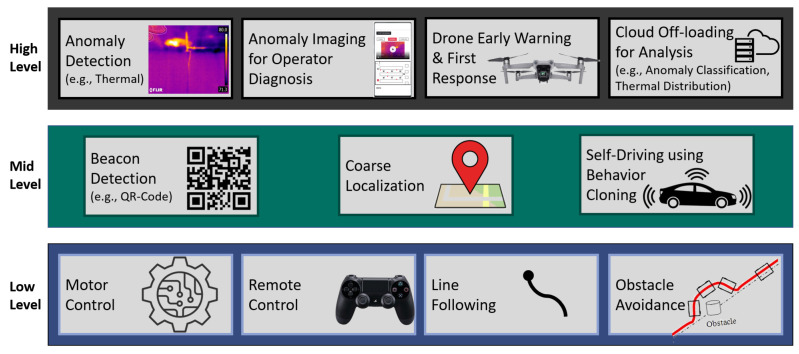
Robot surveyor operating system architecture with modular sub-systems. The architecture is general and adaptable to different applications through the high-level layer.

The central unit of the overall system is an IoT node carried on by our autonomous robot surveyor. The IoT node integrates all sensors needed for inspection. We use modular sub-systems organized in the layered architecture shown in Figure 2 to design our robot surveyor operating system. Communication between all sub-systems, regardless of their level, uses a publish-subscribe pattern. Low-level sub-systems include ones for motor control, remote control, line-following, and obstacle avoidance. Motor control receives command signals from mid-level sub-systems such as self-driving or low-level ones such as remote control and obstacle avoidance, with conflicts resolved through a priority setting. Motor control also publishes robot speed feedback to subscribed sub-systems (e.g., for localization and navigation). Mid-level sub-systems handle localization and behavior cloning and are thus responsible for autonomous driving. High-level sub-systems are application layer systems customizable to the inspection requirements and the industrial environment challenges. For example, since our proposed architecture and robots are tested and validated on the problem of thermal anomalies detection in aluminum factories, we deploy thermal and visual imagers as part of the IoT node sensors. Also, since our testing showed communication networks are inaccessible in the environment, we add to the application layer a messenger drone for early response services (e.g., early alerts). Long-term analyses of anomalies are done by offloading to the cloud. In our application area, this includes the frame rate and resolution adaptive multi-modal (i.e., light and temperature) image registration for the detection and visualization of thermal anomalies. Other analyses can be tailed to the application needs.

### 3.3. Proposed Edge-Fog-Cloud Architecture for Industrial Monitoring

**Figure 3:**
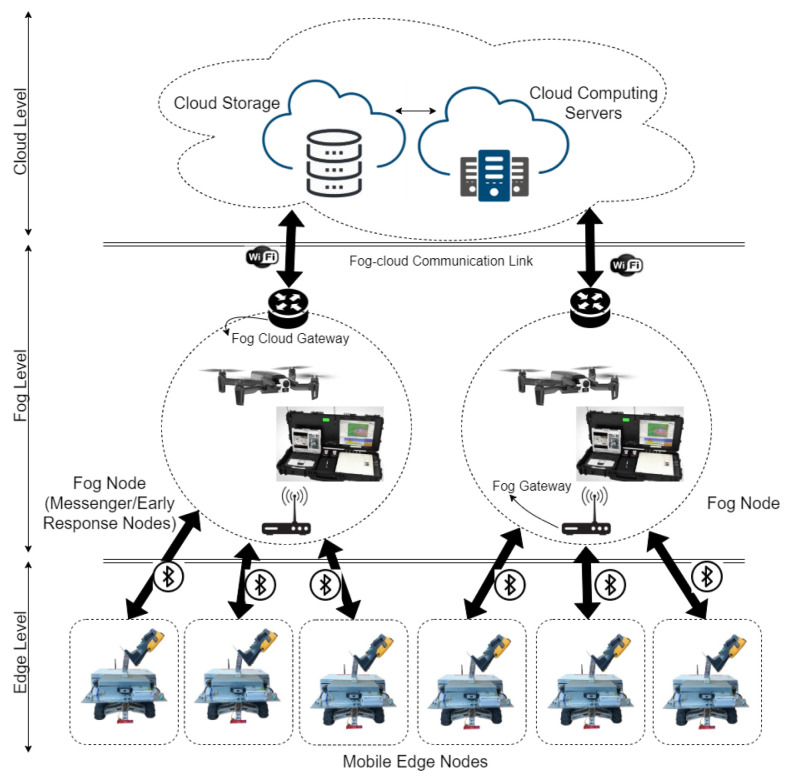
Edge-fog-cloud architecture for the communication needs of autonomous industrial monitoring.

While the IoT nodes carried on the robot surveyors are the central system, the companion drones and the cloud back-end are critical for early response and detailed reporting and analysis. We propose an edge-fog-cloud architecture for autonomous industrial monitoring communication needs to increase response time and reduce surveying costs. We depict the architecture in Figure 3. In our architecture, robot surveyors are mobile edge computing nodes providing surveying and early anomaly detection services, companion drones are fog computing nodes providing early warning and first response services and the back-end is powered by cloud computing nodes providing analysis, storage, and tracking services. What distinguishes our architecture is the AI-powered autonomous nature, or smartness, of our edge and fog nodes, reducing the number of nodes needed to cover the monitored area. As discussed in Section 1, placing fixed IoT nodes to monitor a large industrial area has practicality challenges. Limiting computing to the cloud increases the response latency. Our edge-fog-cloud computing architecture distributes computing closer to the source of the industrial monitoring data reducing latency and improving network utilization.

### 3.4. Thermal Anomalies Detection in Aluminium Factories

**Figure 4:**
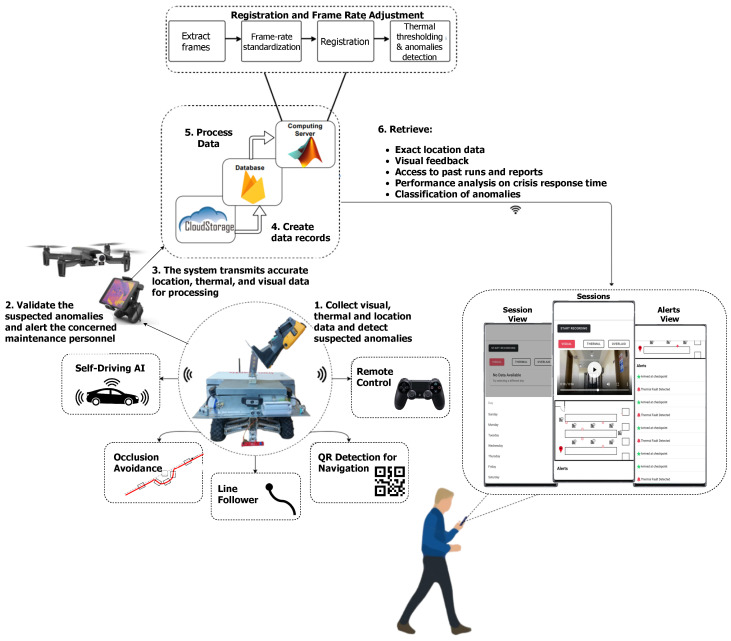
Proposed system architecture.

In Section 3.2 and Section 3.3, we present our proposed architectures designed to address industrial monitoring needs and challenges. In this section, we implement these architectures as we detail our proposed system for thermal anomalies detection in aluminum factories. The overall system diagram is depicted in Figure 4 with numbers indicating the sequence of operations. Our system uses a mobile application to deliver early alerts from the edge and fog layers and detailed reports from the cloud layer to the end-users. The drone is used to reduce the alert time and to initiate automated early response actions if needed (e.g., activate shutdown or cooling routines).

In Section 3.5, we present the robot’s mechanical and electronic design, including shielding design needed to protect the carried IoT node from the harsh environment (e.g., the magnetic field). In Section 3.6, Section 3.7 and Section 3.8, we discuss our proposed self-driving, QR detection navigation, and remote control and robot-preservation sub-systems, respectively. We use a line following sub-system in parts of the environment where visual navigation is not possible (e.g., due to low lighting conditions).

Our robot uses a high frame rate global thermal thresholding to detect anomalies while on survey run. While the robot cannot communicate with the cloud server due to the effects of the environment, it can establish a short-range communication link over Bluetooth to a companion drone as long as a line of sight is available. Once a thermal anomaly is detected, the robot dispatches the drone to the nearest location with access to communication networks to send the thermal and visual images and coarse location data to the user as an early alarm system. The drone can find and join a communication network faster by immediately navigating out of the dead-zone. When the drone leaves the robot to deliver the alert, the connection is lost but is also no longer needed. After the completion of a survey run, recorded thermal and visual videos are uploaded to the cloud. Uploaded videos are processed by a computing server that applies our proposed multi-modal thermal to visual registration to detect and localize thermal anomalies. Analysis reports are delivered to the end user’s mobile application, as explained in Section 3.9.

### 3.5. Robot Body and Magnetic Shielding

**Figure 5:**
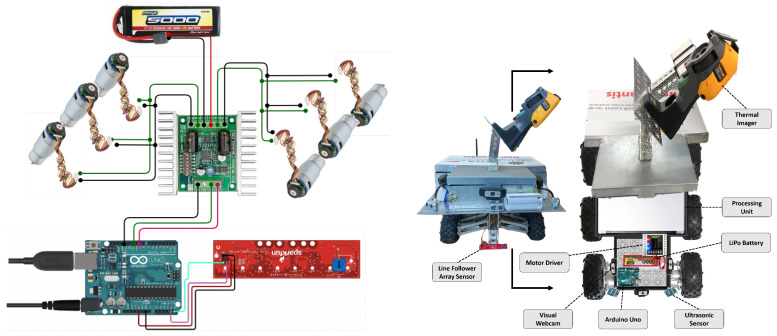
Mechanical and electronic design.

Our AI-powered autonomous robot surveyor is a six-wheeled all-terrain vehicular robot, which consists of an 18” long boxed channel chassis connected to six 5.4” off-road tires. The tires are driven by six 313 rpm ball-bearing precision planetary gear-motors, which allow the robot to navigate tough terrain with ease. The suspension incorporates 4.62” aluminum beams and 130mm, oil-filled, aluminum-bodied shocks. We shield the computing engine, controllers, and sensors from the magnetic field as illustrated in Figure 5. We also added an extension to safely position the line follower sensor array closer to the ground for improved and reliable performance. The robot mechanical and electronic design is shown in Figure 5.

### 3.6. Proposed Self-Driving Deep Architecture for Steering Angle and Speed Regression

We use a behavior-cloning approach to self-driving. An operator remotely controls the robot to complete several runs using real-time video streams as visual feedback. We collect training data by capturing both the speed, the steering angle, and the video signal from all three cameras mounted facing forward, right, and left with a small degree of visual overlap. We then train a deep convolutional neural network to predict the robot’s speed and steering angle. After training, we use the network to autonomously control the robot using real-time images from the three cameras. We cascade the images captured from the camera into a single image.

**Figure 6:**
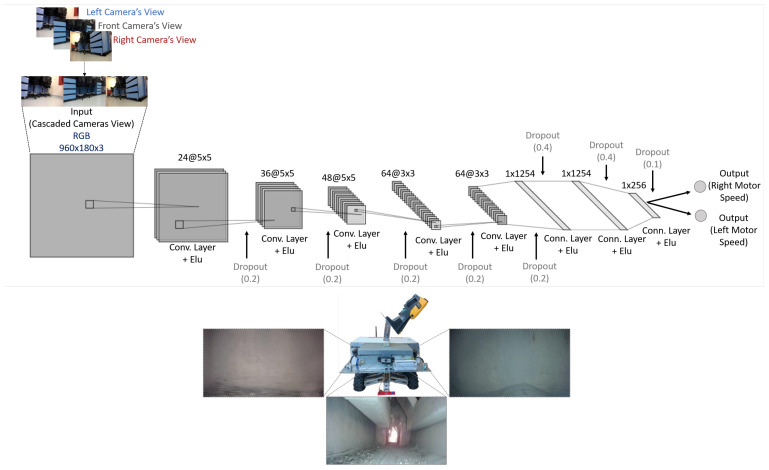
Top: Proposed self-driving deep architecture for real-time speed and steering angle control. Bottom: mounted wide-angle cameras and corresponding views on-site.

Our network, detailed in Figure 6, consists of five convolutional layers with an exponential linear unit (ELU) activation followed by three fully-connected layers. The output layer produces predictions for the right and left DC motor speeds controlled by pulse-width modulation (PWM) signals. Controlling the speed of the motors simultaneously achieves speed and steering angle control. To generalize better and reduce over-fitting, we add dropout layers after every convolutional layer, reduce temporal redundancy by artificially lowering the frame rate, use wide-angle cameras to increase visual details, and capture our videos under varying lighting conditions.

Both the architecture design and the computational power of the robot’s computing unit influence the frame rate and energy needs of the robot. For example, a high frame rate reduces the motor controller sample time and improves stability. We aim to minimize the root mean square error for the PWM levels prediction while simultaneously maximizing the frame rate and battery life. We tune the hyper-parameters using a grid search approach using the lab environment videos and use the tuned parameter for the site environment operations. During training, we optimize the network weights using stochastic gradient descent with adaptive moment estimation.

### 3.7. Robot Surveyor Localization and Navigation Using QR Code Detection

**Figure 7:**
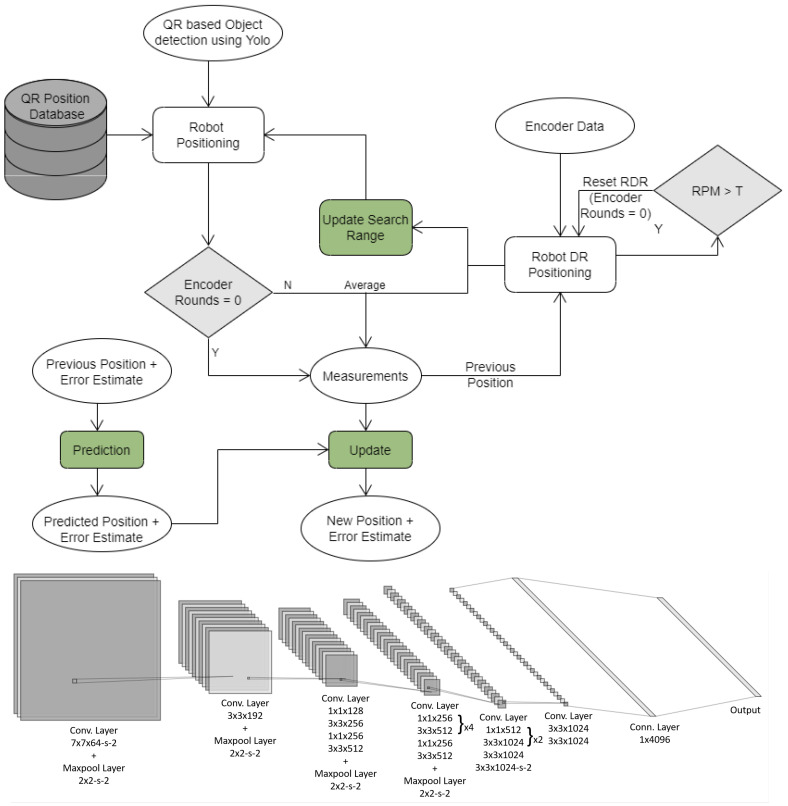
Top: Flow chart of QR-based robot positioning [29] with QR codes instead of radio beacons, round counting instead of steps counting, and the addition of a Kalman filter. Bottom: Used YOLOv2 QR code detector.

We use a modified localization system to the one in ref. [29], as shown in Figure 7. Since the use of radio beacons is impractical due to environmental limitations, we replace them in [29] with visual Quick Response (QR) beacons encoding location data. The QR codes are placed along the surveyed path. The three wide-angle cameras feeding video to the self-driving sub-system are also monitored for QR codes. We use the You Only Look Once (YOLO) object detector in Figure 7 to detect the QR code in the image and decode the absolute location information embedded within. We also replace steps counting in ref. [29] used for dead-reckoning with rotations counting from the DC motors encoders. We consider the output of localization as noisy measurements with Gaussian noise and use a Kalman filter to improve location accuracy. We finally utilize location information to navigate within the environment using a waypoint navigation system.

### 3.8. Remote Control and Collision Avoidance

**Figure 8:**
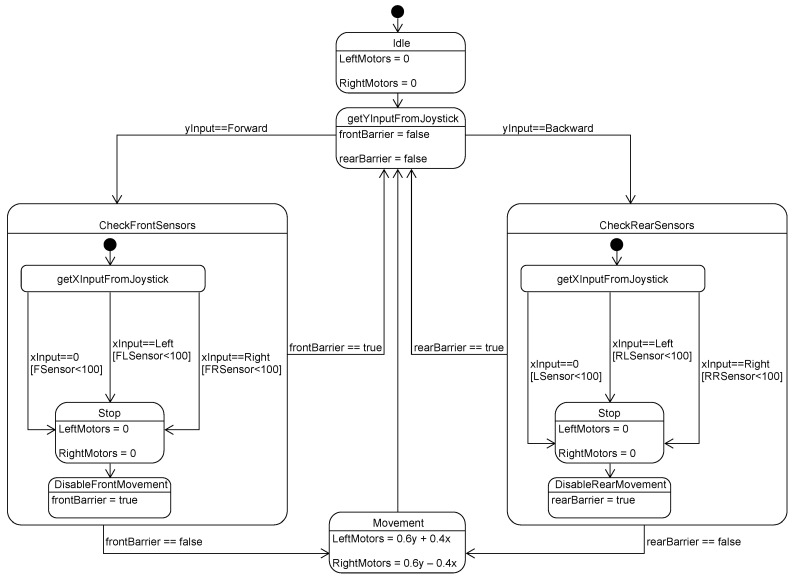
Remote control with collision avoidance UML statechart.

We use a PlayStation controller to transmit control commands to the robot over Bluetooth. We map the controller’s two analog joysticks to the PWM levels of the right and left motor groups. The PWM levels are the ones recorded during training and regressed while self-driving. We use six ultrasonic sensors mounted on the robot to detect obstacles and avoid collisions. The controller unified modeling language (UML) state chart is shown in Figure 8.

### 3.9. Proposed Anomaly Localization Using Thermal to Visual Registration

After a robot surveyor run is complete, thermal and visual videos are offloaded to our cloud server for storage and processing. The thermal imager is sensitive to temperature ranging from −20 °C to 550 °C and captures 9 VGA frames per second. The visual camera capture 29 HD frames per second. The first step of our proposed algorithm is to align the two frames using the capture timestamp by spatiotemporally down-sampling the visible video to match the frame rate and (approximate) resolution of the thermal imager.

We discard color information while registering the two frames and convert the visual frame V(t) from the RGB color-space to gray-scale using the sRGB standard luma coefficients. We represent each pixel in the false-color thermal frame N(t) by its hue angle in the CIE $L^{*}C^{*}h$ color-space with $180^{\circ }\leq h<540^{\circ }$. This particular range of hue angles preserves relative temperature information, i.e., $h_{1}<h_{2}$ iff $T_{1}<T_{2}$, for thermal images using the Iron color-map. We high-pass filter V(t) and N(t) using Sobel and Gaussian ($\sigma^{2}=1\;\mathrm{pixel}$) kernels to compute the gradient magnitude images highlighting object boundaries, $V^{\prime }\left(t\right)$ and $N^{\prime }\left(t\right)$, derived from edges in the visible light and thermal infrared images, respectively.

We estimate a sequence of affine transformations A(t) that aligns $N^{\prime }\left(t\right)$ with $V^{\prime }\left(t\right)$ frame by frame by maximizing mutual information (MI) using

(1) $$\mathrm{MI}\left(A*N^{\prime },V^{\prime }\right)=\sum_{l=0}^{255}\sum_{m=0}^{255}p\left(l,m\right)\log_{2}\left(\frac{p(l,m)}{p_{N^{\prime }}\left(l\right)p_{V^{\prime }}\left(m\right)}\right),$$

where $A*N^{\prime }$ is the warped thermal gradient magnitude, *l* is the temperature level (remapped to 0–255), *m* is the intensity level, p(l,m) is the joint probability distribution, and $p_{N^{\prime }}\left(l\right)$ and $p_{V^{\prime }}\left(m\right)$ are the marginal probability distributions of the thermal and visual frames, respectively. We initialize A(t) for each frame with a pure translation aligning the frames’ geometric centers and find the optimal transformation ${\mathrm{argmax}}_{A}\mathrm{MI}$ using the anisotropic (1 + 1)-ES algorithm [30].

We smooth the transformations to reduce jitter in the registered videos. Let each frame-specific affine transformation matrix A(t) be the parameterized six-element vector

(2) $$a\left(t\right)=\left(\Delta_{x},\Delta_{y},h,s_{x},s_{y},\sin\theta \right),$$

where $\left(\Delta_{x},\Delta_{y}\right)$ are translations, *h*, $s_{x}$, and $s_{y}$ together determine shear and non-uniform scaling, and θ is the angle of rotation. We smooth a(t) elementwise using local cubic polynomial fitting and generate the smoothed affine transformations $A_{s}\left(t\right)$. The corresponding transformed frames $N_{V}\left(t\right)=A_{s}\left(t\right)*N\left(t\right)$ then smoothly track the down-sampled V(t), and are interpolated in *t* and resized to match the original video resolution and frame rate. We detect and mark anomalies by thermal thresholding of the blended video and generate a report with the robot location and blended video temporally aligned. Our cloud server delivers the report directly to the end user’s mobile application.

## 4. Validation and Results

In this section, we present the testing results for each of the robot’s sub-systems. We also validate our proposed architectures by discussing the end to end results of deploying our system on-site at an aluminum factory in the United Arab Emirates.

### 4.1. Objective Results for Proposed Self-Driving Network

**Figure 9:**
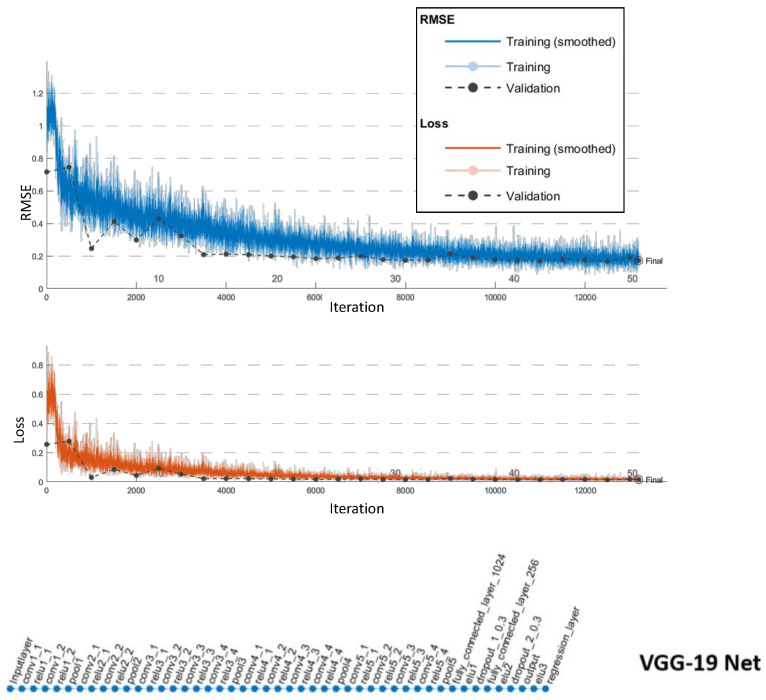
VGG-19: configured with a learning rate of $10^{-1}$, piece-wise scheduling, and 50 Epochs. Trained in 989 min and a final validation RMSE of 0.17.

**Figure 10:**
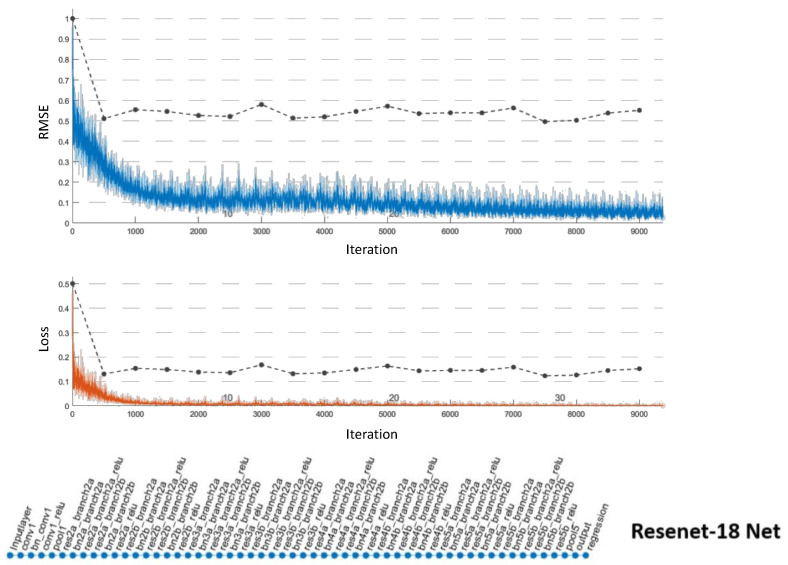
Resnet-18: configured with a learning rate of $10^{-1}$, piece-wise scheduling, and 50 Epochs. Terminated early in 77 min and a final RMSE of 0.55 with clear signs of over-fitting.

**Figure 11:**
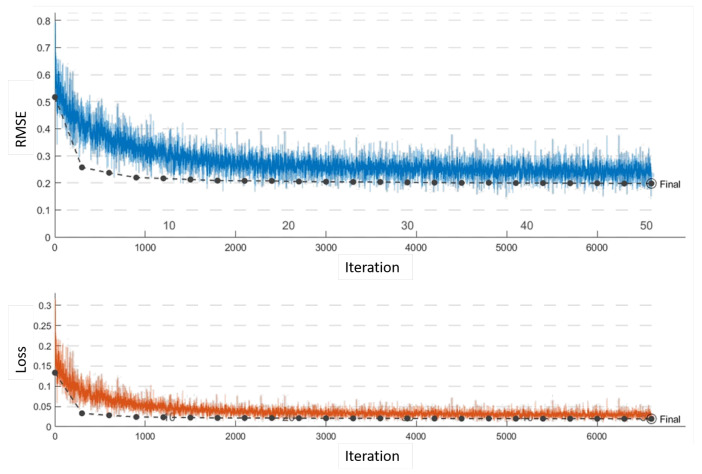
Proposed network: configured with a learning rate of $10^{-1}$, piece-wise scheduling, and 50 Epochs. Completed 50 Epochs in 23% of the VGG-19’s time and final RMSE of 0.19.

To validate our self-driving quantitatively, we trained our proposed, VGG-19, and Resnet-18 networks on the same training data set. We first trained on 5000 images and tested on an equal number in the lab environment collected from 10 complete runs. We then used transfer learning and further optimized all networks using an additional 5000 images collected from site runs. We also added 5000 site images to our testing set. We optimized the hyper-parameters using a grid search approach for all networks. The VGG-19 regression network delivered the lowest validation root mean squared error (RMSE) at 0.17, took approximately 989 min to train, and delivered a maximum frame rate of 22 frames per second while in operation. The results for the VGG-19 networks are shown in Figure 9. We terminated the Resenet-18 training early due to repeated signs of over-fitting, which can be observed from Figure 10. Our proposed network performance is shown in Figure 11. It delivered an RMSE close to that of VGG-19 at 0.19 in 224 min of training time. Our network had a higher frame rate of 65 frames per second. All training and testing were carried out on the same single GPU computer as the central processing unit for the robot.

### 4.2. Subjective Results for Proposed Self-Driving Network

**Figure 12:**
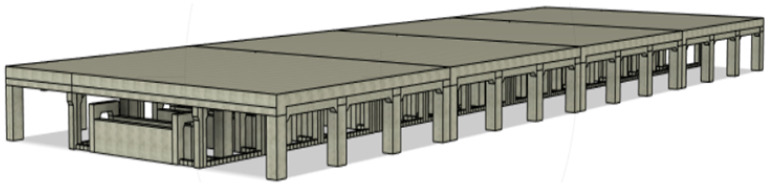
Model of the basement of the potline.

**Figure 13:**
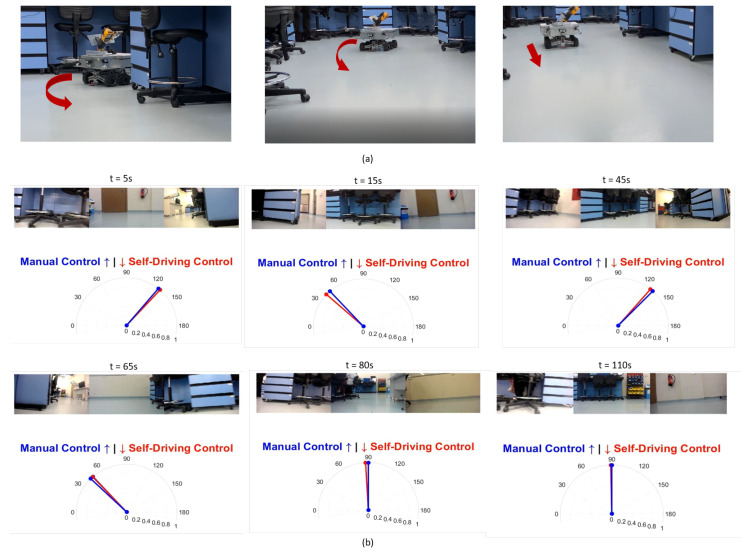
[Lab test run 1] Self-Driving in the lab environment results: Figures in (**a**) show the robot turning around the center bench autonomously then moving forward after turning. Figures in (**b**) represent the robot’s view and the true vs. regressed steering angles.

**Figure 14:**
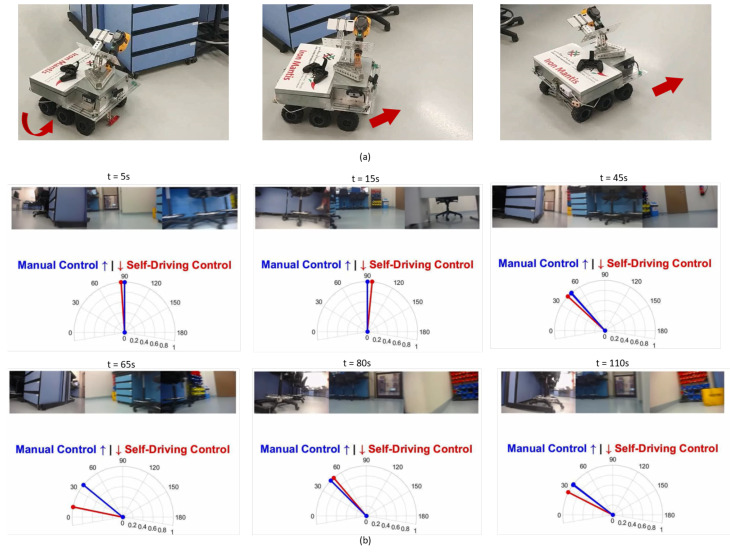
[Lab test run 2] Self-Driving in the lab environment results: Figures in (**a**) show the robot turning around the side bench autonomously then moving forward after turning. Figures in (**b**) represent the robot’s view and the true vs. regressed steering angles.

**Figure 15:**
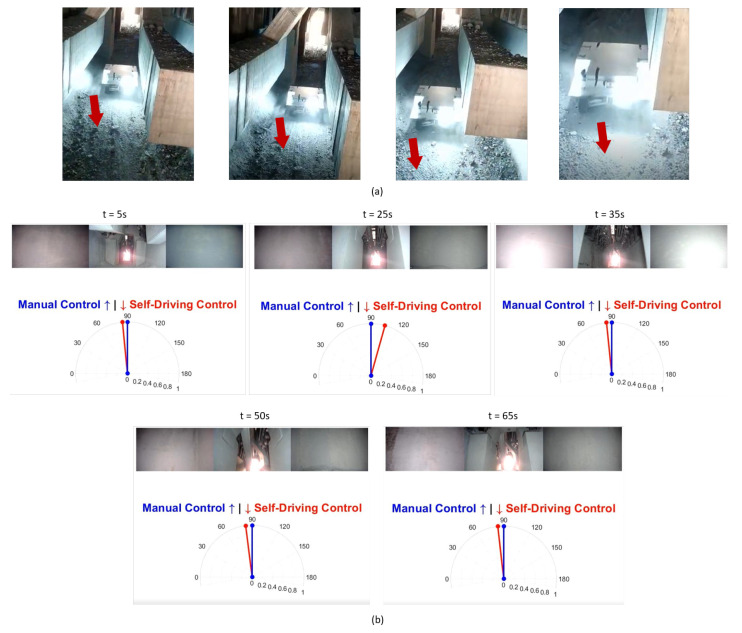
[Site test run 1] Self-Driving on-site results: Figures in (**a**) show the robot going into the environment autonomously. Figures in (**b**) represent the robot’s view and the true vs. regressed steering angles.

**Figure 16:**
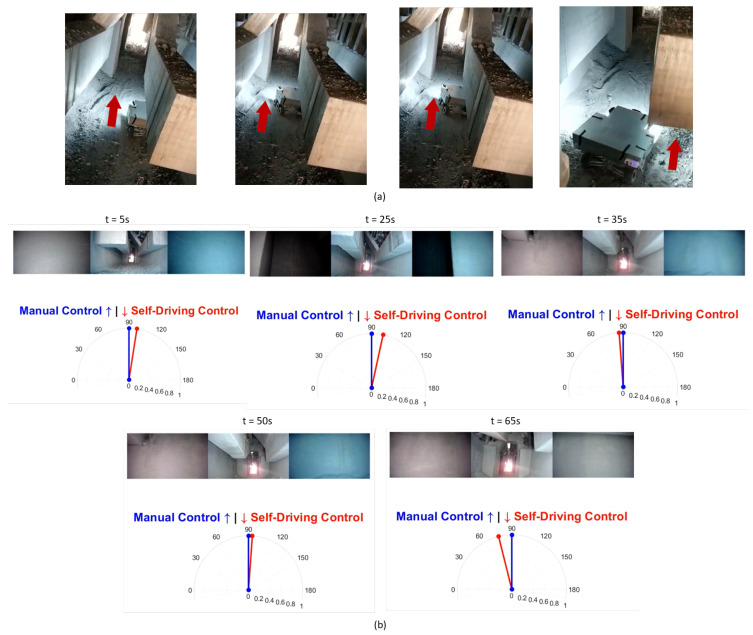
[Site test run 2] Self-Driving on-site results: Figures in (**a**) show the robot going out of the environment autonomously. Figures in (**b**) represent the robot’s view and the true vs. regressed steering angles.

We tested our robot surveyor in both the lab and site environments and it was able to complete all 20 runs without collisions. Figure 12 shows the model of the potline’s basement and depicts the path conditions and obstacles configuration. The potline consists of several parallel pots with narrow spacing and paths for operators to carry regular manual measurements. The robot path usually suffers debris from the aluminum production process, bricks, and sandstones. We visually compare the true and regressed steering angles over time including both the robot and observer viewpoints in Figure 13 and Figure 14 for two test runs in the lab environment and Figure 15 and Figure 16 for two test runs in the site environment, respectively. Our robot cloned the remote operator behavior and faithfully reproduced the same control signals.

### 4.3. QR Detection Localization Results

**Figure 17:**
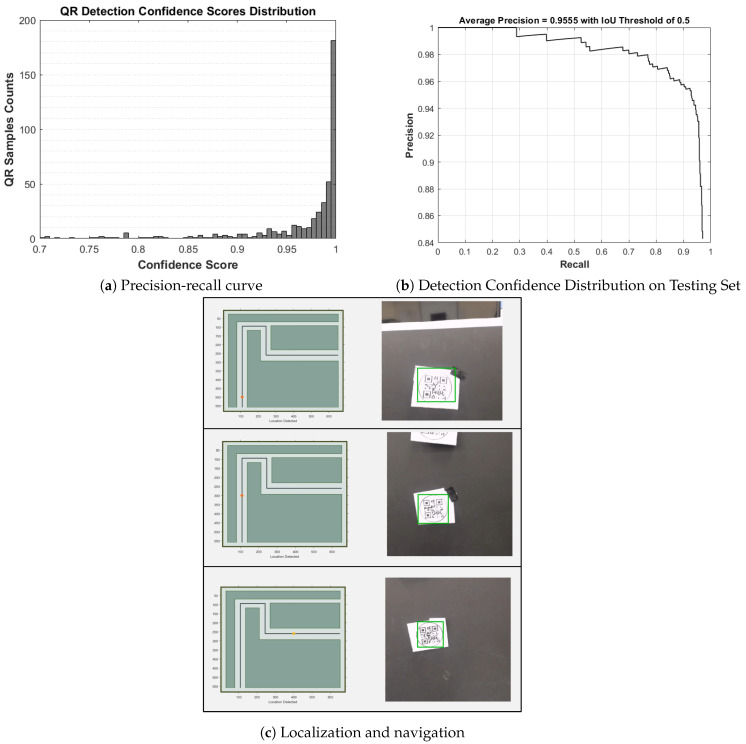
Detection performance for the Quick Response (QR) robot localization and navigation.

We generated 5000 QR images with random location data to train our YOLO QR object detector and used 500 images to test the detector’s performance using an intersection over union (IoU) threshold of 0.5. In Figure 17a, we show the histogram of confidence scores for the 500 test images with a clear bias to high confidence scores. The detector was able to recognize 90% of the QR codes in the testing set with an average precision of 0.96 and the precision vs. recall curve shown in Figure 17b. We observed that QR detection is more precise on-site than in the lab environment, which is attributed to the site’s higher visual homogeneity. Figure 17c shows a sequence of QR codes detections, decoding, and use in navigation.

### 4.4. Obstacle Avoidance Response Time

**Figure 18:**
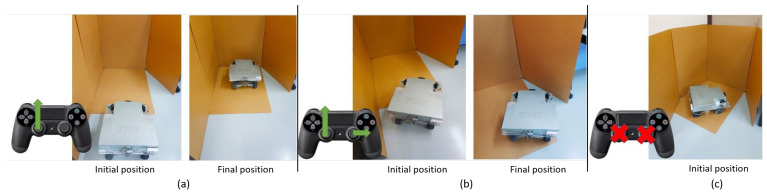
Obstacle avoidance algorithm testing results shows the robot stopping as an obstacle is detected by the (**a**) front middle sensor, (**b**) front right sensor, and (**c**) front and rear sensors.

To test the self-preservation sub-system, we designed seven testing scenarios in the lab environment. In each scenario, we measured the detection response time. Obstacles can be detected from the front middle sensor, front right sensor, front left sensor, rear middle sensor, rear left sensor, rear right sensor, and front and rear sensors at the same time. We detail three testing scenarios below:

- Scenario 1: An obstacle is detected from the front middle sensor. When the distance retrieved from the ultrasonic sensor centered at the front is small, indicating an obstacle exists in front of the robot, the robot moves to the stop state. After that, all motion is blocked except backward, as illustrated in Figure 18a.
- Scenario 2: An obstacle is detected from the front right sensor. When an obstacle exists on the front right side, the robot stops, and motion is restricted to backward Figure 18b.
- Scenario 3: Obstacles are detected from the front and rear sensors. As depicted in Figure 18c, when the front and backward sensors detect obstacles in rare cases, the robot stops, all motion commands are blocked.

The detection and response times of each scenario are tabled in Table 1. Our system can respond within 0.3 s of being close to an obstacle by 60 cm from the front and rear sides and 30 cm from the left and right sides as measured in the lab.

**Figure 19:**
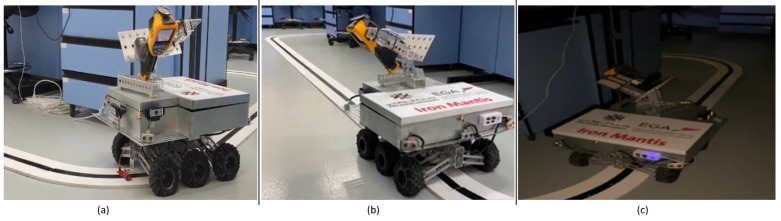
The Line Follower algorithm results in the lab environment: (**a**) shows the robot following the track and turning around the center bench while (**b**) shows the robot while it continues after turning and moves forward. (**c**) shows the results of testing the line follower algorithm under different lighting conditions.

Our robot reverts to line-following when lighting conditions are too low for self-driving as demonstrated in Figure 19.

### 4.5. Visual Results of Thermal Anomaly Segmentation and Visualization

**Figure 20:**
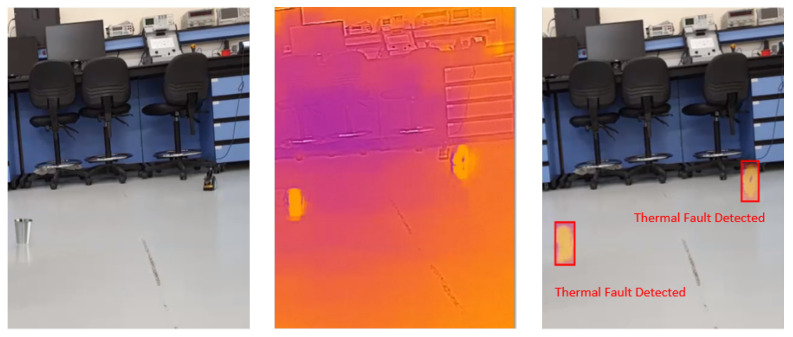
Thermal anomalies detection in lab environment 1.

**Figure 21:**
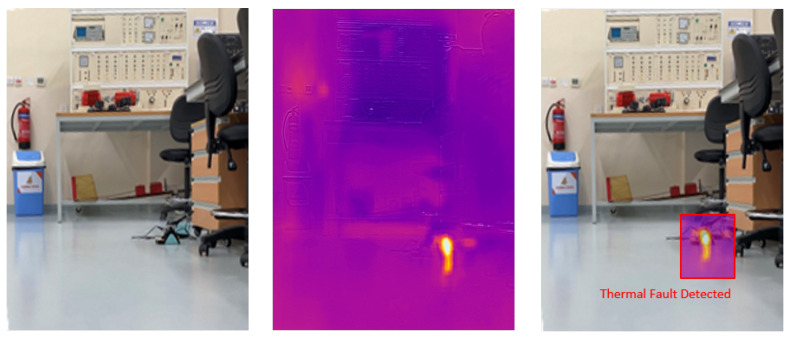
Thermal anomalies detection in lab environment 2.

**Figure 22:**
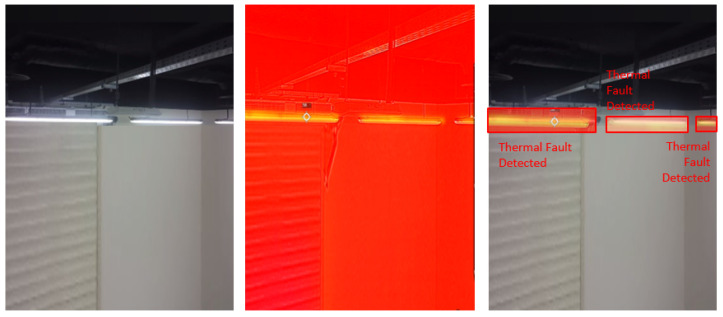
Thermal anomalies detection in lab environment 3.

**Figure 23:**
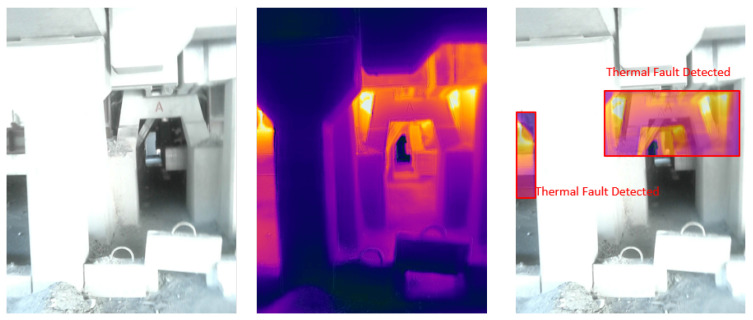
Thermal anomalies detection on-site for test run 1.

**Figure 24:**
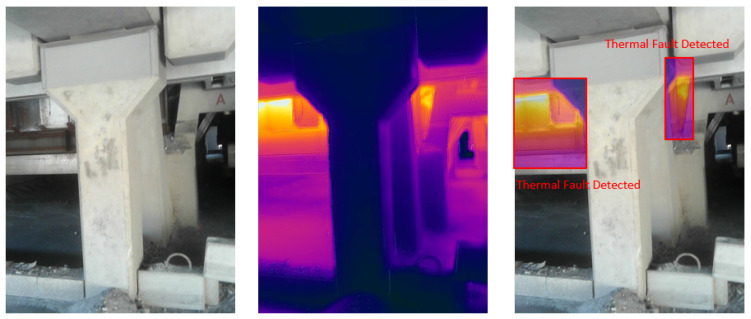
Thermal anomalies detection on-site for test run 2.

**Figure 25:**
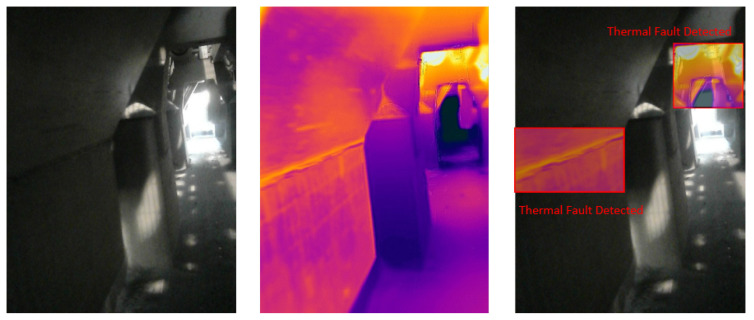
Thermal anomalies detection on-site for test run 3.

**Figure 26:**
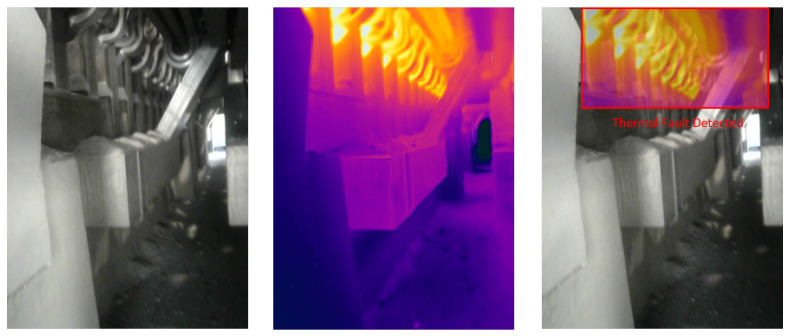
Thermal anomalies detection on-site for test run 4.

**Figure 27:**
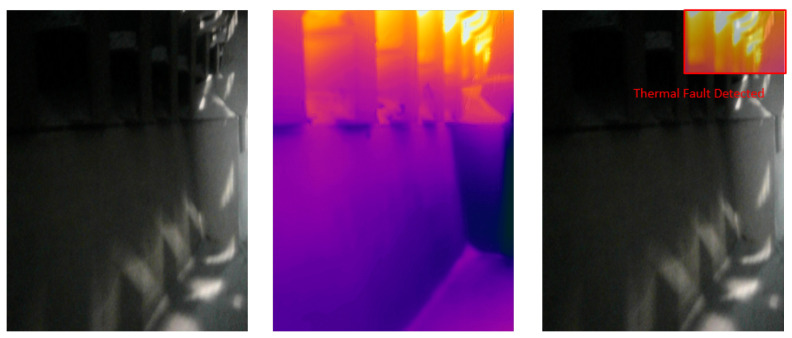
Thermal anomalies detection on-site for test run 5.

We tested our thermal to visual registration and anomaly detection algorithm in three different lab environments and five runs in the site environment and concluded that it can accurately warp the thermal frame and align it to the visual frame despite being captured at different resolutions and frame rates. Figure 20, Figure 21 and Figure 22 illustrate the results of testing in the labs, and Figure 23, Figure 24, Figure 25, Figure 26 and Figure 27 on-site. In the lab setting, we heat objects to a target temperature guided by real-time measurements. We then configure our thermal detectors to detect this target temperature range. These surfaces may not be as hot as the anomaly range since it is difficult to replicate conditions leading to an anomaly, but they are hot enough to test our system. On-site, we manually measure the temperature of hot surfaces using handheld thermal cameras. In all cases, we detected and segmented the thermal anomalies. To produce sufficient testing video sequences, we lowered the thermal threshold for testing and re-calibrated the color-map to create more testing scenarios. During operations, the sensors and thresholds can be re-calibrated to meet the specific needs of the process.

### 4.6. End to End Integration Testing and Validation

**Figure 28:**
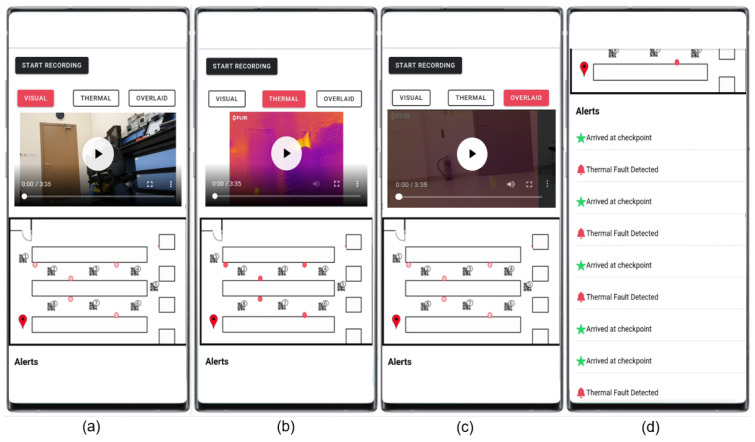
Mobile app displaying alerts and showing the localization and the ability to switch between the different views to be able to analyze thermal anomalies: (**a**) shows the visual view, (**b**) shows the thermal view while (**c**) shows the registered view. (**d**) shows the alerts received.

We performed end to end integration testing by placing hot objects in five different locations in the lab. Our robot surveyor completed its lab run then uploaded the thermal and visual videos and location data to the cloud server. All five hot objects were detected and localized on the lab environment map, as shown in Figure 28. Early warnings from the drone were delivered in a short response time (less than 1 s) along with location data as can be seen in Figure 28d.

The user can switch between the visual, thermal, or blended views to analyze the thermal anomaly.

### 4.7. Computational Complexity and Response Time Testing

The most computationally demanding sub-system during a survey run is self-driving. We compare the number of layers, iterations until convergence, training time, validation RMSE, and frame rate for our self-driving network to VGG-19 and Resnet-19 in Table 2.

Our proposed network outperforms VGG-19 with respect to training and testing complexities but slightly lags in terms of validation RMSE. Nevertheless, our self-driving tests confirm our proposed network’s ability to regress the control signals for the robot accurately to autonomously complete all runs. Thermal to visual registration on the cloud happens offline at a frame rate of 3 frames per second.

We define alert time to be the time between the robot surveyor detecting a thermal anomaly while on a run until the initial alert with images and location information is delivered to the end user’s mobile application. This performance parameter is mostly affected by the length of the run and the location of the robot at the anomaly detection time since it influences the time the messenger drone needs to find a communication network. If the communication network is available during a run, the alert time is practically insignificant for our application needs. Our average alert time in the lab environment is measured at 6.7 s. We define response time to be the time between the robot detecting an anomaly until the full run report with complete thermal anomaly visualization and location information becomes available for the end-user. Response time is affected by the length of the run and the location of the robot. Our average response time in the lab environment is measured to be 16.03 min. Both these measures are far less than the time it takes a human surveyor to detect and report the anomaly.

### 4.8. Initial Setup Stage for New Environments

In this Section, we discuss the initial setup needed to use our system in a different environment. We design the mechanical and electronic systems to operate under challenging conditions. The robot has an A-arm style suspension and nearly 5” of independent wheel travel. It also has 313 rpm ball-bearing precision planetary gearmotors giving enough torque to turn 5.4” off-road tires allowing it to overcome obstacles. The suspension incorporates 4.62” aluminum beams and our 130 mm, oil-filled, aluminum bodied shocks. The motor driver supplies the DC motors with 25A for each group with a peak current of 50A per group for a few seconds. The shielding protects from high magnetic fields and operating temperatures. The initial setup stage requires additional training of the self-driving sub-system for improved performance. This can be accomplished by putting the robot in remote control mode and driving it in the environment for 5–10 runs. The robot automatically records the videos and controls and uploads them to the cloud. The self-driving architecture is trained on the newly collected data. The robot downloads the trained model and is ready to go. This additional training does not mean the robot trains from scratch. We still use the trained network from our lab and environment videos. The additional training serves to optimize the network to the new environment. We also re-calibrate the thermal camera and set the detectors thresholds to target the expected anomalies range, much like tuning a bandpass filter. Finally, QR codes need to be fixed around the environment for localization. The same initial setup stage was used in the three lab environments and the on-site environment.

## 5. Conclusions

In this paper, we propose an edge-fog-cloud architecture for autonomous industrial monitoring of thermal anomalies. At the edge layer, robot surveyors carrying IoT nodes scan the environment’s temperature in real-time for thermal anomalies in the absence of communication networks to report alerts. The robots use our proposed deep network running at 65 frames per second with a root mean squared error of 0.19, 0.02 less than the deeper VGG-19 network, and three times faster. If anomalies are detected, a mobile fog node in the form of a companion drone is dispatched looking for connectivity to deliver an early alert in 6.7 s on average. When a run is complete, the robot uploads thermal and visual videos and location information to a cloud back-end server. We use a proposed thermal to visual registration algorithm to maximize mutual derivative information and spatio-temporally align and localize thermal anomalies. End-users receive detailed reports, including the aligned video with thermal anomalies localized in 16.03 min, far sooner than the human survey time. We tested our proposed architecture in both the lab and onsite and concluded it efficiently monitors a sizeable industrial area despite its challenging characteristics. Our system’s limitations are survey path segments with low lighting conditions affecting self-driving and the lack of connectivity in the environment, which we have addressed by shifting autonomous driving to rely on line-following in these segments and adding a companion drone to deliver alerts in a short time. Further research is needed to address these limitations better.

## Figures and Tables

**Table 1:** Obstacles detection and response times.

Scenario	Obstacle Location	Obstacle Detection Time (s)	Obstacle Response Time (s)
Scenario 1	front center	0.00350	0.29350
Scenario 2	front right	0.00146	0.23146
Scenario 3	front left	0.00146	0.22146
Scenario 4	rear center	0.00350	0.21350
Scenario 5	rear right	0.00146	0.24146
Scenario 6	rear left	0.00146	0.26146
Scenario 7	front and rear	0.00350	0.20350

**Table 2:** Comparison of the different network architectures.

Network Architecture	Number of Layers	Iterations	Training Time (mins)	Validation RMSE	Frame Rate
VGG-19	47	13,200	989	0.17	22
Resenet-18	65	-	77	0.55	-
Proposed architecture	28	6600	228	0.19	65
